# Adjuvant Docetaxel–Cyclophosphamide Versus Anthracycline–Taxane Chemotherapy in Stage I–III HR-Positive/HER2-Negative Breast Cancer: A Real-World Propensity Score Overlap-Weighted Cohort Study

**DOI:** 10.3390/jcm15218568

**Published:** 2026-11-03

**Authors:** Perihan Perkin, Derya Sarikaya, Hilal Karakas, Ugur Ozberk, Burak Bilgin, Efnan Algin, Oznur Bal, Didem Sener Dede, Gokhan Ucar

**Affiliations:** Department of Medical Oncology, Ankara Bilkent City Hospital, 06800 Ankara, Türkiye; drderyasarikaya@gmail.com (D.S.); dnc_hilal@hotmail.com (H.K.); ugurozberk94@gmail.com (U.O.); dr_bbilgin@yahoo.com (B.B.); efnanalgin@gmail.com (E.A.); dr_ozn@yahoo.com (O.B.); ddmsener@yahoo.com (D.S.D.); gokhanucar_1@hotmail.com (G.U.)

**Keywords:** early breast cancer, HR-positive/HER2-negative, adjuvant chemotherapy, docetaxel–cyclophosphamide, anthracycline–taxane, propensity score overlap weighting, real-world evidence

## Abstract

**Background/Objectives:** The optimal adjuvant chemotherapy regimen remains uncertain for some patients with hormone receptor (HR)-positive/HER2-negative early breast cancer, particularly regarding the incremental benefit of anthracyclines. We compared real-world outcomes with docetaxel-plus-cyclophosphamide (TC) and anthracycline–taxane chemotherapy. **Methods:** This retrospective single-center cohort included patients with stage I–III HR-positive/HER2-negative breast cancer who underwent surgery and received adjuvant TC or anthracycline–taxane chemotherapy between 2019 and 2025. Patients receiving neoadjuvant systemic therapy were excluded. Disease-free survival (DFS) was the primary outcome and overall survival (OS) the secondary outcome. Propensity score overlap weighting adjusted for measured baseline differences was used; the absolute difference in weighted 3-year DFS was the primary adjusted measure. **Results:** Among 361 patients, 201 received TC and 160 anthracycline–taxane chemotherapy. The anthracycline–taxane group had higher baseline clinicopathologic risk. Median follow-up was 39.0 months. Twelve DFS events occurred (TC, *n* = 3; anthracycline–taxane, *n* = 9). Unadjusted 3-year DFS was 99.0% versus 96.6% (*p* = 0.096), and 3-year OS was 99.4% versus 99.1% (*p* = 0.725). The overlap-weighted analysis included 346 patients and achieved adequate covariate balance. Weighted 3-year DFS was 99.2% with TC and 96.5% with anthracycline–taxane chemotherapy, an absolute difference of 2.68 percentage points (95% CI, −1.81 to 10.03). Sensitivity analysis yielded similar results (2.70 percentage points; 95% CI, −1.76 to 9.87). **Conclusions:** No statistically significant difference in 3-year DFS was demonstrated after adjustment. Limited events and relatively short follow-up preclude conclusions regarding equivalence or noninferiority. These findings support individualized adjuvant chemotherapy selection and warrant confirmation in larger cohorts with longer follow-up.

## 1. Introduction

Hormone receptor (HR)-positive/HER2-negative early breast cancer is clinically heterogeneous, and adjuvant systemic treatment is increasingly individualized according to clinicopathologic risk and, when available, genomic information [1]. Endocrine therapy remains the cornerstone of treatment, whereas chemotherapy is generally considered for patients with higher-risk disease. Even after the decision to administer chemotherapy has been made, however, the optimal regimen and the incremental value of anthracycline exposure remain uncertain in patients without unequivocally high-risk features.

Anthracycline–taxane-based chemotherapy is an established adjuvant option for higher-risk HER2-negative breast cancer [2]. A large patient-level meta-analysis by the Early Breast Cancer Trialists’ Collaborative Group showed an overall reduction in recurrence with anthracycline-containing taxane regimens compared with taxane regimens without anthracyclines, although the magnitude of benefit varied by treatment schedule, and no significant reduction was observed in trials comparing sequential anthracycline–taxane schedules with docetaxel plus cyclophosphamide (TC) [2]. Any potential efficacy advantage must be weighed against long-term toxicities, including cardiotoxicity and the rare risk of therapy-related myeloid malignancies [2,3].

TC emerged as an anthracycline-free option after US Oncology 9735 demonstrated superior disease-free survival (DFS) and overall survival (OS) with four cycles of TC versus four cycles of doxorubicin plus cyclophosphamide [4]. Subsequent randomized comparisons of TC with anthracycline–taxane chemotherapy largely evaluated six cycles of TC (TC6) rather than four cycles [5,6]. In the ABC trials, TC6 failed to meet the prespecified noninferiority criterion versus anthracycline–taxane therapy in high-risk HER2-negative disease [5], whereas the pooled PlanB/SUCCESS C analysis found no significant overall DFS or OS advantage for anthracycline–taxane chemotherapy, with exploratory benefit mainly in pN2–pN3 disease [6]. A 2024 meta-analysis similarly found no overall DFS or OS difference, although anthracycline–taxane therapy was associated with improved DFS in patients with four or more involved lymph nodes [7]. Ten-year WSG PlanB data also showed similar long-term outcomes with TC6 and EC-T in predominantly pN0–pN1 HR-positive/HER2-negative disease [8]. Thus, direct comparative evidence specifically addressing four-cycle TC remains limited.

Treatment selection in routine practice further complicates comparisons because anthracycline–taxane regimens are preferentially used in patients with less favorable clinicopathologic features, creating confounding by indication [9]. Moreover, most randomized comparative evidence has evaluated six-cycle TC, whereas direct evidence regarding four-cycle TC in contemporary HR-positive/HER2-negative practice remains limited. We therefore compared DFS and OS between adjuvant TC and anthracycline–taxane chemotherapy in patients with stage I–III HR-positive/HER2-negative breast cancer treated in routine clinical practice, with a focus on the real-world use of predominantly four-cycle TC, using propensity score overlap weighting to account for measured baseline differences. The primary research question was whether 3-year DFS differed between the two treatment strategies after adjustment for measured baseline differences. This study was designed as a comparative-effectiveness analysis and not as a noninferiority study.

## 2. Materials and Methods

### 2.1. Study Design and Patient Population

This retrospective, single-center cohort study included patients with stage I–III hormone receptor (HR)-positive/HER2-negative breast cancer who underwent definitive breast surgery and received adjuvant chemotherapy at Ankara Bilkent City Hospital between 2019 and 2025. Patients who had received neoadjuvant systemic therapy were excluded because the study was designed to compare postoperative adjuvant chemotherapy strategies using baseline risk characteristics derived from definitive surgical pathology.

Eligible patients had histologically confirmed HR-positive/HER2-negative breast cancer, no distant metastatic disease at diagnosis, definitive surgical treatment, and receipt of either docetaxel plus cyclophosphamide (TC) or an anthracycline–taxane-based regimen. Patients were excluded if they did not meet the HR-positive/HER2-negative criteria, received a chemotherapy regimen outside these treatment groups, had insufficient clinical or follow-up data, or received systemic treatment following an ipsilateral local recurrence rather than as part of the index adjuvant treatment course. Treatment selection was made in routine clinical practice at the discretion of the treating physicians.

The study was approved by the Ankara Bilkent City Hospital No. 1 Medical Research Scientific and Ethics Evaluation Committee (TABED; approval date: 25 February 2026; reference no. TABED 1-26-2239). Informed consent was not required because of the retrospective nature of the study, in accordance with institutional policy.

### 2.2. Treatment Definitions and Data Collection

The TC regimen consisted of docetaxel 75 mg/m^2^ plus cyclophosphamide 600 mg/m^2^ every 3 weeks, generally for four cycles. Anthracycline–taxane chemotherapy consisted of four cycles of doxorubicin 60 mg/m^2^ plus cyclophosphamide 600 mg/m^2^ followed by weekly paclitaxel 80 mg/m^2^, typically for 12 doses. The AC component was administered according to either a dose-dense (ddAC+T) or conventional (AC+T) schedule. Treatment delivery was characterized using the recorded number of chemotherapy cycles, dose reductions, early discontinuation, and completion of the planned regimen. Individual administered doses and infusion dates were not systematically abstracted into the analytic dataset, precluding reliable calculation of relative dose intensity. Switching between TC and anthracycline–taxane regimens was not recorded as a separate variable and therefore could not be reliably quantified.

Clinical, pathological, treatment, and follow-up data were retrospectively abstracted from institutional records. Baseline variables included age, menopausal status, Eastern Cooperative Oncology Group (ECOG) performance status, histology, grade, ER and PR expression, HER2 status, Ki-67, tumor size, pathologic T and N categories, number of positive lymph nodes, lymphovascular invasion, and perineural invasion.

HR positivity was defined as ER and/or PR expression in ≥1% of tumor cell nuclei by immunohistochemistry [10]. All patients in the final cohort had ER-positive disease. HER2-negative disease was defined as an immunohistochemistry score of 0 or 1+, or 2+ without HER2 gene amplification by in situ hybridization, in accordance with ASCO/CAP criteria [11].

Pathologic staging was based on the anatomic staging system of the eighth edition of the American Joint Committee on Cancer. Histology, tumor grade, and Ki-67 from diagnostic biopsy and surgical specimens were recorded separately when available. Treatment-related variables included breast and axillary surgery, chemotherapy completion, dose reduction, early discontinuation, radiotherapy, and endocrine therapy. Follow-up data included recurrence, recurrence site and date, last follow-up, vital status, and date of death.

### 2.3. Outcomes and Follow-Up

The primary outcome was disease-free survival (DFS), defined as the time from definitive breast surgery to the first documented local or distant recurrence or death from any cause, whichever occurred first. Patients without an event were censored at the date of last follow-up.

Overall survival (OS) was a secondary outcome and was defined as the time from definitive breast surgery to death from any cause. Patients alive at last follow-up were censored at the date of their most recent clinical assessment. Recurrences were categorized as local or distant according to the documented site of disease.

Outcome information was retrospectively obtained from institutional electronic medical records, including oncology outpatient clinic notes and radiological imaging reports. Recurrence dates were determined from the first documented evidence of recurrence, while dates of death and last follow-up were obtained from available clinical records. Available follow-up information was additionally cross-checked through the national e-Nabız electronic health record platform. A deceased-patient access restriction displayed by the platform was used as supplementary information regarding vital status rather than as a source of death dates. All 361 patients included in the final study cohort had recorded surgery dates, last follow-up dates, and vital status information.

### 2.4. Statistical Analysis

Continuous variables were summarized using medians and ranges, and categorical variables were summarized using frequencies and percentages. Between-group comparisons were performed using the Mann–Whitney U test for continuous variables and Pearson’s chi-square or exact tests, as appropriate, for categorical variables. All tests were two-sided, with *p* < 0.05 considered statistically significant.

Unadjusted DFS and OS were estimated using the Kaplan–Meier method and compared with the log-rank test. Hazard ratios (HRs) and 95% confidence intervals (CIs) for anthracycline–taxane chemotherapy relative to TC were estimated using Cox proportional hazards models. Median follow-up was estimated using the reverse Kaplan–Meier method.

Because treatment allocation was nonrandomized, propensity score overlap weighting was used to reduce confounding by measured baseline characteristics [12]. The propensity score, defined as the probability of receiving anthracycline–taxane chemotherapy, was estimated using multivariable logistic regression including age, year of diagnosis, menopausal status, tumor size, pathologic nodal burden, biopsy grade, biopsy Ki-67, biopsy histology, and lymphovascular invasion. Covariates were selected based on their clinical relevance to treatment selection and recurrence risk. Year of diagnosis was included to account for potential temporal changes in treatment selection during the study period. Genomic recurrence scores were not systematically available, and comorbidity variables were not included in the propensity-score model. Nodal burden was categorized as N0, N1/N1mi, or N2–N3, and menopausal status was categorized as premenopausal versus post/perimenopausal. Age, year of diagnosis, tumor size, and Ki-67 were entered as continuous variables.

Overlap weights were defined as 1 minus the propensity score for patients receiving anthracycline–taxane chemotherapy and as the propensity score for patients receiving TC. Covariate balance was assessed using absolute standardized mean differences (SMDs), with an absolute SMD < 0.10 considered adequate balance [13].

The primary propensity score analysis used complete cases for the covariates included in the model, without imputation. ECOG performance status was not included in the primary propensity-score model because of missing data and the resulting reduction in analyzable sample size. Effective sample size (ESS) was calculated separately for each treatment group as the squared sum of the overlap weights divided by the sum of squared weights. The total ESS was calculated as the sum of the group-specific effective sample sizes.

In the overlap-weighted cohort, weighted Kaplan–Meier methods were used to estimate DFS. The primary estimand was the absolute difference in 3-year DFS between TC and anthracycline–taxane chemotherapy in the propensity-score overlap population, expressed as TC minus anthracycline–taxane chemotherapy. Because of the limited number of events and declining numbers at risk with longer follow-up, 3-year DFS was selected as the primary adjusted time point. Ninety-five percent CIs were estimated using 5000 treatment-stratified nonparametric bootstrap resamples, with re-estimation of the propensity score and overlap weights in each resample.

A sensitivity analysis repeated the overlap-weighting procedure using tumor grade, Ki-67, and histology from the surgical specimen instead of the corresponding biopsy-derived variables while retaining the remaining covariates unchanged. Confidence intervals were estimated using the same bootstrap procedure as in the primary analysis.

An additional exploratory sensitivity analysis was performed among patients with complete ECOG and biopsy-based covariate data. ECOG performance status (0, 1, or 2) was incorporated as a categorical variable into the primary propensity-score model while retaining all other covariates unchanged. Propensity scores and overlap weights were re-estimated, and the absolute difference in 3-year DFS was calculated using the same bootstrap procedure as in the primary analysis. Missing ECOG values were not imputed.

To assess potential guarantee-time bias associated with defining DFS from the date of surgery, an exploratory 90-day postoperative landmark sensitivity analysis was performed. Patients were eligible for this analysis if they had complete primary propensity-score covariate data, had initiated adjuvant chemotherapy within 90 days after surgery, and remained free of DFS events at the landmark. Follow-up was measured from postoperative day 90. Propensity scores and overlap weights were re-estimated in the landmark cohort using the primary model covariates. The absolute difference in 3-year DFS from the landmark was estimated using weighted Kaplan–Meier methods, with 95% confidence intervals obtained from 5000 treatment-stratified bootstrap resamples.

Given the limited number of DFS events, an exploratory parsimonious multivariable Cox proportional-hazards model was additionally fitted as a sensitivity analysis. To minimize overfitting, the model was restricted to treatment group, maximum tumor size (entered per 10 mm increase), and pathologic nodal burden (N0, N1/N1mi, or N2–N3). This analysis was considered complementary to the primary overlap-weighted analysis, and its estimates were interpreted cautiously because of the limited number of outcome events.

Statistical analyses were performed using Python 3.13 with NumPy (https://numpy.org), SciPy (https://scipy.org), statsmodels (https://www.statsmodels.org), and scikit-learn (https://scikit-learn.org). The official software websites were accessed on 25 October 2026.

OpenAI ChatGPT was used to provide programming assistance for exploratory sensitivity analyses; the analyses were based on the original clinical dataset, and the authors retained responsibility for verification and interpretation.

## 3. Results

### 3.1. Study Population and Baseline Characteristics

**Figure 1:**
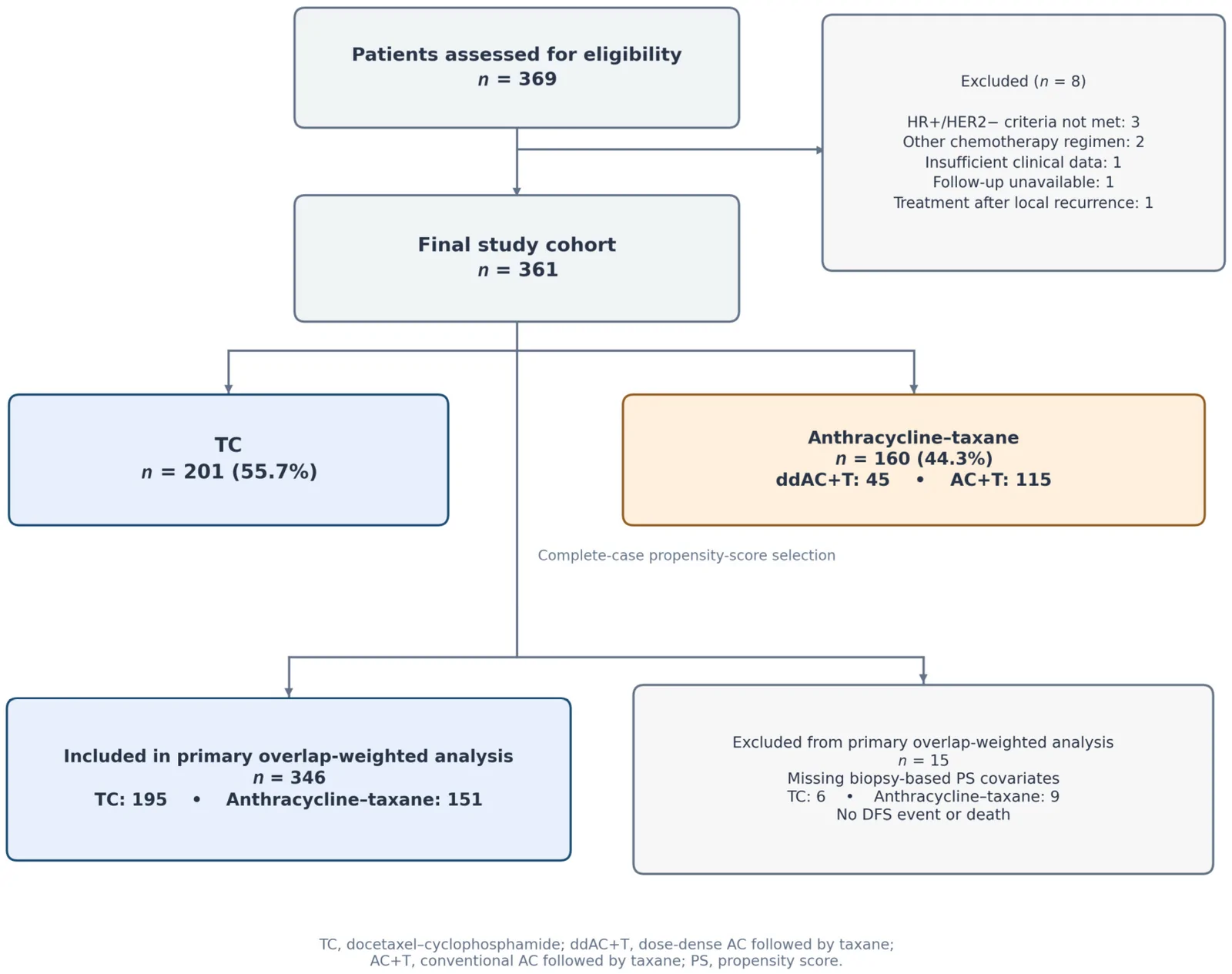
Study flow diagram. Flow of patients through eligibility assessment and classification according to the adjuvant chemotherapy regimen received. TC, docetaxel plus cyclophosphamide; ddAC+T, dose-dense doxorubicin/cyclophosphamide followed by a taxane; AC+T, doxorubicin/cyclophosphamide followed by a taxane. The primary overlap-weighted analysis included 346 complete-case patients; 15 patients were excluded because of missing biopsy-based propensity-score covariates, and none experienced a DFS event or death.

A total of 369 patients were assessed for eligibility; 8 were excluded because the tumor did not meet the HR-positive/HER2-negative eligibility criteria (*n* = 3), the chemotherapy regimen was outside the prespecified treatment groups (*n* = 2), clinical data were insufficient (*n* = 1), follow-up data were unavailable (*n* = 1), or systemic treatment had been initiated following an ipsilateral local recurrence rather than as part of the index adjuvant treatment course (*n* = 1). The final cohort comprised 361 patients, of whom 201 (55.7%) received TC and 160 (44.3%) received anthracycline–taxane chemotherapy, including ddAC+T (*n* = 45) and AC+T (*n* = 115) (Figure 1).

Baseline clinicopathologic risk was higher in the anthracycline–taxane group. These patients were younger, had larger tumors and greater nodal burden, and more frequently had stage III disease and lymphovascular invasion. Stage III disease was present in 30.6% of the anthracycline–taxane group versus 3.0% of the TC group. Biopsy tumor grade also differed between groups, whereas biopsy Ki-67 did not. Baseline characteristics are summarized in Table 1.

### 3.2. Treatment Patterns and Delivery

Chemotherapy delivery was similar between groups. Dose reduction occurred in 10.0% of patients receiving TC and 12.5% receiving anthracycline–taxane chemotherapy (*p* = 0.501), early discontinuation in 3.5% and 5.6% (*p* = 0.441), and planned treatment completion in 96.0% and 94.4%, respectively (*p* = 0.467). Among patients receiving TC, 189 received four cycles, four received six cycles, and eight received fewer than four cycles. Among patients receiving anthracycline–taxane chemotherapy, 142 had documented administration of four AC cycles followed by 12 weekly paclitaxel doses. The median interval between definitive surgery and chemotherapy initiation was 34 days (interquartile range, 23–47 days) overall, 35 days in the TC group, and 32 days in the anthracycline–taxane group. Chemotherapy was initiated within 90 days after surgery in 352 of 361 patients. No DFS events were documented before chemotherapy initiation.

Breast and axillary surgical procedures differed between groups, consistent with the greater baseline disease burden in patients receiving anthracycline–taxane chemotherapy. Adjuvant radiotherapy was administered to 88.1% and 91.3% of patients in the TC and anthracycline–taxane groups, respectively (*p* = 0.389). Endocrine therapy was administered to nearly all patients (99.5% vs. 99.4%; *p* = 1.000), and endocrine treatment strategies did not differ significantly between groups (*p* = 0.085). Treatment characteristics are summarized in Table 2.

### 3.3. Unadjusted Survival Outcomes

**Figure 2:**
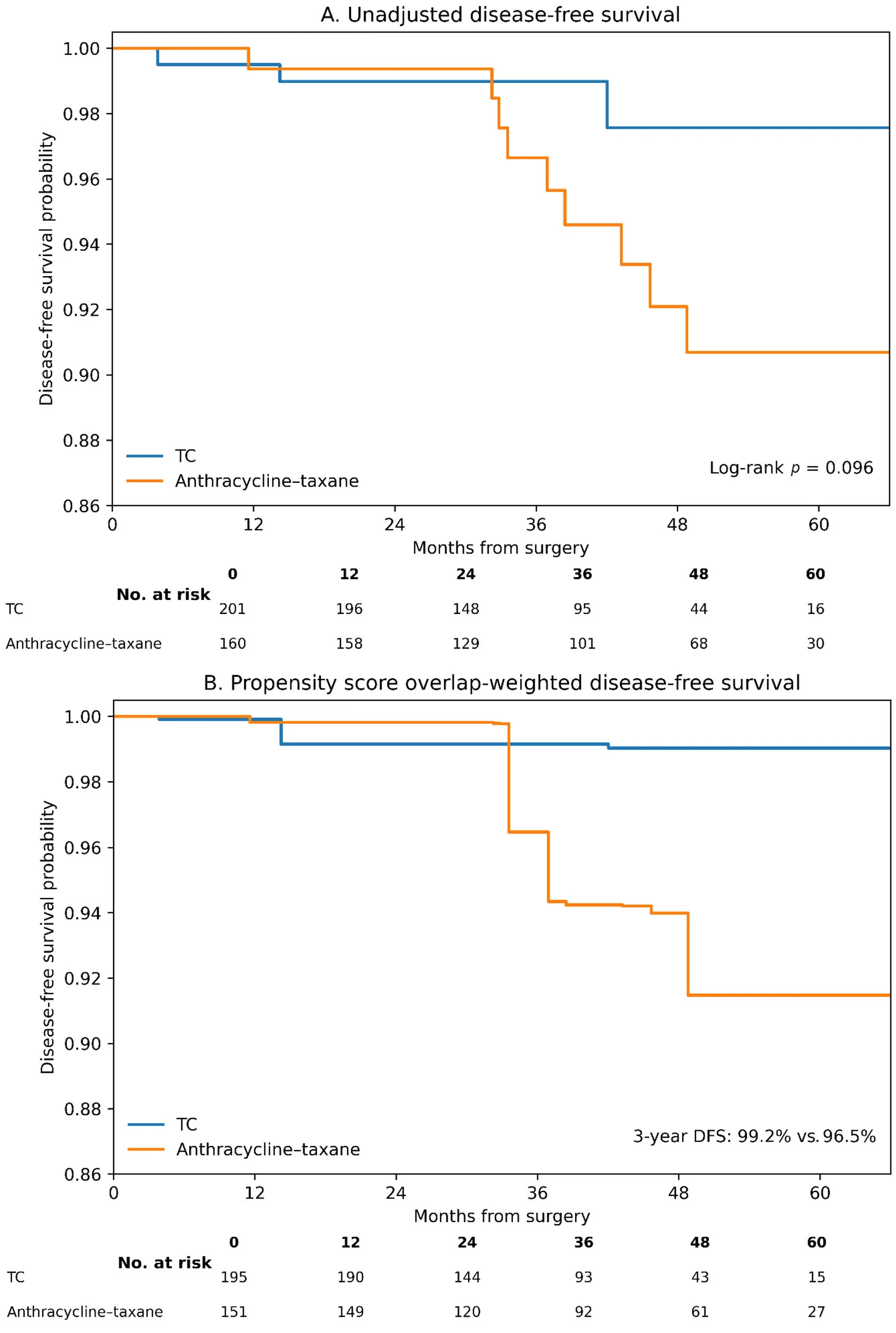
Disease-free survival according to adjuvant chemotherapy regimen. (**A**) Unadjusted Kaplan–Meier estimates of disease-free survival in the overall study cohort. (**B**) Propensity score overlap-weighted disease-free survival estimates in the primary complete-case cohort. Numbers at risk in panel B represent the observed, unweighted numbers of patients remaining under follow-up in the corresponding treatment groups. DFS, disease-free survival; TC, docetaxel plus cyclophosphamide.

Median follow-up, estimated using the reverse Kaplan–Meier method, was 39.0 months overall, 35.0 months for TC, and 44.8 months for anthracycline–taxane chemotherapy. Surgery dates, last follow-up dates, and vital status information were available for all 361 patients included in the final study cohort. Twelve DFS events occurred: 3 among 201 patients receiving TC and 9 among 160 receiving anthracycline–taxane chemotherapy. Overall, eight recurrences were documented, including six distant and two local recurrences. Estimated 3-year DFS was 99.0% with TC and 96.6% with anthracycline–taxane chemotherapy; corresponding 5-year estimates were 97.6% and 90.7%. The difference was not statistically significant (log-rank *p* = 0.096), with an unadjusted HR of 2.90 (95% CI, 0.78–10.77) for anthracycline–taxane chemotherapy versus TC (Table 3; Figure 2A).

Five deaths occurred, including two in the TC group and three in the anthracycline–taxane group. Three-year OS was 99.4% and 99.1%, respectively, and 5-year OS was 98.0% and 96.6%. No significant difference in OS was observed (log-rank *p* = 0.725; HR, 1.38; 95% CI, 0.23–8.34). Median DFS and OS were not reached. The unadjusted OS curve is shown in Supplementary Figure S3.

### 3.4. Propensity Score Overlap-Weighted Analysis

**Figure 3:**
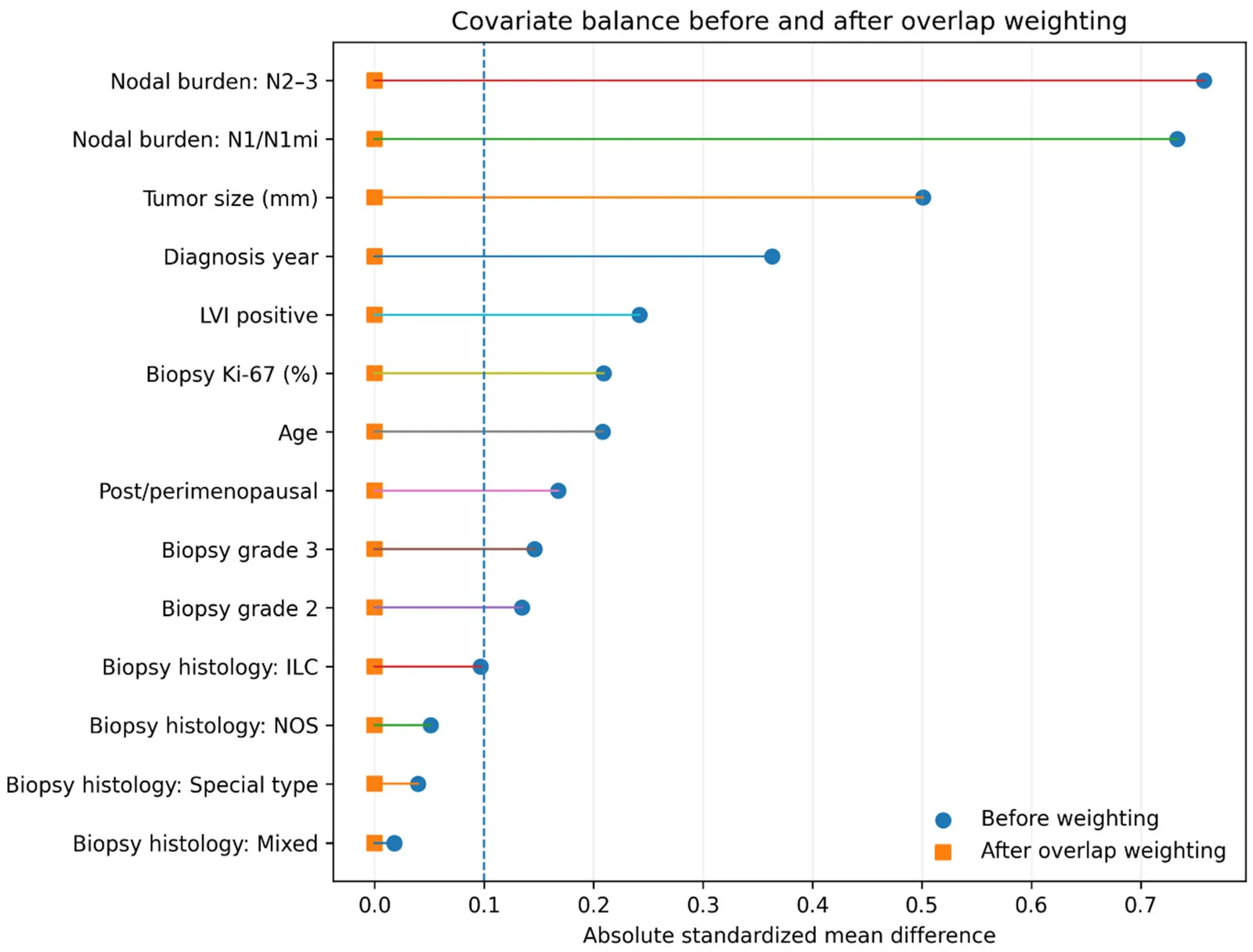
Covariate balance before and after propensity score overlap weighting. Absolute standardized mean differences for covariates included in the primary propensity score model are shown before and after overlap weighting. An absolute standardized mean difference < 0.10 was considered indicative of adequate covariate balance.

The primary propensity score analysis included 346 complete cases (TC, *n* = 195; anthracycline–taxane, *n* = 151). Fifteen patients were excluded because of missing biopsy-derived covariates; none experienced a DFS event or death. The estimated effective sample sizes after overlap weighting were 81.4 for TC and 80.2 for anthracycline–taxane chemotherapy, with a total ESS of approximately 161.6. Substantial baseline imbalance was present before weighting, whereas overlap weighting achieved excellent balance across all modeled covariates, with the maximum absolute SMD < 0.001 after weighting (Figure 3; Supplementary Table S1). Propensity-score distributions before and after weighting are shown in Supplementary Figures S1 and S2.

In the overlap-weighted cohort, estimated 3-year DFS was 99.2% with TC and 96.5% with anthracycline–taxane chemotherapy. The absolute TC-minus-anthracycline–taxane difference was 2.68 percentage points (95% CI, −1.81 to 10.03), with no statistically significant difference after adjustment for measured baseline characteristics (Table 3; Figure 2B).

Results were consistent in the sensitivity analysis using surgical-specimen grade, Ki-67, and histology. Among 360 patients, estimated 3-year DFS was 99.3% with TC and 96.6% with anthracycline–taxane chemotherapy, corresponding to an absolute difference of 2.70 percentage points (95% CI, −1.76 to 9.87) (Supplementary Table S2).

In the additional exploratory sensitivity analysis incorporating ECOG performance status, 323 complete-case patients were included (TC, *n* = 195; anthracycline–taxane, *n* = 128), with 11 DFS events. Estimated 3-year DFS was 99.7% with TC and 95.5% with anthracycline–taxane chemotherapy, yielding an absolute difference of 4.14 percentage points (95% CI, −0.99 to 13.44). Although the point estimate remained numerically in favor of TC, the confidence interval included zero. Missing ECOG data were confined to the anthracycline–taxane group, limiting the interpretation of this exploratory analysis (Supplementary Table S4).

In the exploratory 90-day postoperative landmark analysis, 337 patients were included (TC, *n* = 193; anthracycline–taxane, *n* = 144), with 12 DFS events. After overlap weighting, estimated 3-year DFS from the landmark was 99.1% with TC and 94.0% with anthracycline–taxane chemotherapy, yielding an absolute difference of 5.14 percentage points (95% CI, −1.06 to 14.34). The confidence interval included zero, and this analysis was interpreted cautiously because of the small number of events and the restriction to patients who had initiated chemotherapy within 90 days after surgery (Supplementary Table S4).

In the exploratory parsimonious multivariable Cox analysis, adjustment for maximum tumor size and pathologic nodal burden substantially attenuated the treatment effect estimate. The adjusted HR for anthracycline–taxane chemotherapy versus TC was 1.32 (95% CI, 0.25–7.06; *p* = 0.745), compared with an unadjusted HR of 2.90 (95% CI, 0.78–10.77). Given the small number of DFS events, this analysis was considered exploratory, and the wide confidence interval should be interpreted cautiously (Supplementary Table S3).

## 4. Discussion

In this real-world cohort of patients with stage I–III HR-positive/HER2-negative breast cancer, both adjuvant TC and anthracycline–taxane chemotherapy were associated with high early DFS despite marked differences in baseline clinicopathologic risk. After propensity score overlap weighting, estimated 3-year DFS was 99.2% with TC and 96.5% with anthracycline–taxane chemotherapy, corresponding to an absolute difference of 2.68 percentage points (95% CI, −1.81 to 10.03). This difference was not statistically significant, and findings were consistent in the sensitivity analysis using surgical-specimen tumor characteristics. The limited number of events and relatively short follow-up constrain the precision of the comparison and assessment of longer-term outcomes. The confidence interval crossed zero and encompassed possible differences in both directions; therefore, the absence of statistical significance cannot establish equivalence or noninferiority between the regimens.

These findings are broadly consistent with randomized and pooled evidence suggesting that the incremental benefit of anthracycline exposure may vary according to baseline recurrence risk. In the pooled PlanB/SUCCESS C analysis, no significant overall DFS or OS advantage was observed with anthracycline–taxane chemotherapy over TC6, although exploratory analyses suggested greater benefit in patients with pN2–pN3 disease [6]. A 2024 meta-analysis similarly found no significant overall DFS or OS difference, while anthracycline–taxane therapy was associated with improved DFS among patients with four or more involved lymph nodes [7]. Ten-year WSG PlanB data also demonstrated similar long-term outcomes with TC6 and EC-T in predominantly pN0–pN1 HR-positive/HER2-negative disease [8]. These observations are relevant to our cohort, in which anthracycline–taxane chemotherapy was preferentially administered to patients with greater disease burden.

The substantial baseline imbalance between treatment groups illustrates the challenge of confounding by indication in observational treatment comparisons. Patients receiving anthracycline–taxane chemotherapy had larger tumors, greater nodal burden, more stage III disease, and more frequent lymphovascular invasion, reflecting routine selection of more intensive regimens for patients perceived to have higher recurrence risk [9]. Accordingly, the numerical separation in the unadjusted DFS curves cannot be interpreted independently of treatment-selection bias. Overlap weighting achieved excellent balance across modeled baseline covariates, and no statistically significant difference in 3-year DFS was demonstrated after adjustment. Consistent with this interpretation, the treatment HR was attenuated from 2.90 in the unadjusted analysis to 1.32 after parsimonious adjustment for tumor size and nodal burden, although the wide confidence interval resulting from the limited number of events precludes a precise estimate of the treatment effect.

Treatment selection must also consider the potential long-term toxicity of anthracyclines, including cardiomyopathy and heart failure [14,15]. When the expected absolute oncologic benefit of anthracycline exposure is small or uncertain, limiting anthracycline exposure may therefore be clinically relevant. However, our findings should not be interpreted as supporting routine replacement of anthracycline–taxane chemotherapy with TC, particularly in patients with substantial nodal burden or other high-risk features [6,7]. An additional consideration is the number of TC cycles. Much of the randomized comparative evidence evaluated TC6, whereas four-cycle TC is used in contemporary practice and remains a guideline-supported adjuvant option [16]. A propensity-score-matched real-world study from Alberta reported similar long-term outcomes with four-cycle TC and anthracycline–taxane chemotherapy in HER2-negative, node-negative disease [17], although generalizability to node-positive or higher-risk populations is uncertain. A large Ontario population-based analysis further demonstrated substantial heterogeneity in outcomes according to regimen and hormone receptor status [9]. Our study therefore contributes additional real-world evidence in an HR-positive/HER2-negative population treated predominantly with four-cycle TC.

Several limitations should be acknowledged. The retrospective, single-center design remains susceptible to residual and unmeasured confounding despite overlap weighting. Because follow-up was based on routine clinical practice rather than a study-mandated surveillance schedule, differences in surveillance intensity between treatment groups cannot be excluded. Furthermore, the retrospective ascertainment of recurrence and survival outcomes may be susceptible to incomplete event capture and loss-to-follow-up bias. Because the primary DFS analysis used surgery as the time origin although chemotherapy was initiated postoperatively, guarantee-time bias remains a potential limitation. An exploratory 90-day landmark analysis was performed to investigate this issue; however, restriction to patients who remained event-free and initiated chemotherapy by day 90 may itself introduce selection bias, and the landmark results reflect a different follow-up window. The small number of DFS events and deaths limited statistical precision, and the median follow-up of 39 months is relatively short for HR-positive/HER2-negative breast cancer, in which recurrence risk persists beyond 5 years [18]. Accordingly, the 5-year survival estimates should be interpreted cautiously because relatively few patients remained at risk at 60 months. Furthermore, overlap weighting targets patients with overlapping treatment probabilities; therefore, these findings may not be generalizable to the broader population of patients with very high-risk disease, particularly those with extensive nodal involvement who were infrequently treated with TC in our cohort. Genomic recurrence scores were not systematically available and therefore could not be incorporated into treatment-selection adjustment; such assays may refine chemotherapy decisions in selected patients [19], although access and reimbursement vary across health-care systems [20]. Comorbidity measures were also not included in the propensity-score model, representing an additional potential source of residual confounding. Although the ECOG-adjusted sensitivity analysis yielded qualitatively similar findings, missing ECOG data occurred exclusively in the anthracycline–taxane group, and complete-case restriction may have introduced additional selection bias. The lack of systematically collected infusion-level dosing and treatment-switching data also prevented formal assessment of relative dose intensity and regimen changes. Strengths include detailed patient-level clinicopathologic, treatment, endocrine-therapy, and recurrence data, adjustment for marked baseline treatment-selection differences, and consistent findings in the surgical-pathology sensitivity analysis.

## 5. Conclusions

In conclusion, no statistically significant difference in 3-year DFS was demonstrated between adjuvant TC and anthracycline–taxane chemotherapy after adjustment for measured baseline differences. Given the limited number of events and relatively short follow-up, these findings should not be interpreted as evidence of equivalence between the regimens. They support individualized treatment selection that considers baseline recurrence risk and the potential benefits and long-term toxicities of anthracycline exposure. Longer follow-up and larger comparative cohorts are needed to better define patients who may be appropriate candidates for anthracycline-sparing strategies.

## Figures and Tables

**Table 1:** Baseline clinicopathologic characteristics of the study cohort.

Characteristic	Overall (*n* = 361)	TC (*n* = 201)	Anthracycline–Taxane (*n* = 160)	*p* Value
**Demographic and clinical characteristics**				
Age at diagnosis, years, median (range)	53.6 (24.8–84.7)	55.3 (25.8–84.7)	51.8 (24.8–79.2)	0.037
Menopausal status, *n* (%)				0.057
Premenopausal	136 (37.7)	68 (33.8)	68 (42.5)	
Postmenopausal	221 (61.2)	129 (64.2)	92 (57.5)	
Perimenopausal	4 (1.1)	4 (2.0)	0 (0.0)	
ECOG performance status, *n* (%)				0.783
0	153 (45.5)	89 (44.3)	64 (47.4)	
1	172 (51.2)	106 (52.7)	66 (48.9)	
2	11 (3.3)	6 (3.0)	5 (3.7)	
**Biopsy tumor characteristics**				
Biopsy histology, *n* (%)				0.885
IDC	18 (5.1)	9 (4.5)	9 (5.8)	
ILC	29 (8.2)	18 (9.0)	11 (7.1)	
NOS/NST	298 (84.2)	166 (83.4)	132 (85.2)	
Mixed	5 (1.4)	3 (1.5)	2 (1.3)	
Special type	4 (1.1)	3 (1.5)	1 (0.6)	
Biopsy grade, *n* (%)				0.027
Grade 1	67 (19.2)	47 (23.9)	20 (13.2)	
Grade 2	243 (69.6)	132 (67.0)	111 (73.0)	
Grade 3	39 (11.2)	18 (9.1)	21 (13.8)	
ER expression, %, median (range)	95.0 (10.0–100.0)	95.0 (10.0–100.0)	95.0 (10.0–100.0)	0.330
PR expression, %, median (range)	80.0 (0.0–100.0)	80.0 (0.0–100.0)	80.0 (0.0–100.0)	0.473
Ki-67, %, median (range)	20.0 (1.0–90.0)	20.0 (1.0–90.0)	20.0 (1.0–90.0)	0.079
**Pathologic disease characteristics**				
Pathologic TNM stage, *n* (%)				<0.001
IA	62 (17.2)	59 (29.4)	3 (1.9)	
IB	5 (1.4)	2 (1.0)	3 (1.9)	
IIA	143 (39.6)	108 (53.7)	35 (21.9)	
IIB	96 (26.6)	26 (12.9)	70 (43.8)	
IIIA	35 (9.7)	6 (3.0)	29 (18.1)	
IIIB	2 (0.6)	0 (0.0)	2 (1.2)	
IIIC	18 (5.0)	0 (0.0)	18 (11.2)	
Maximum tumor size, mm, median (range)	25.0 (0.0–130.0)	22.0 (0.0–80.0)	30.0 (0.0–130.0)	<0.001
Positive lymph nodes, *n*, median (range)	1.0 (0.0–36.0)	0.0 (0.0–7.0)	1.0 (0.0–36.0)	<0.001
Lymphovascular invasion, *n* (%)				0.017
Absent	181 (50.1)	112 (55.7)	69 (43.1)	
Present	180 (49.9)	89 (44.3)	91 (56.9)	
Perineural invasion, *n* (%)				0.582
Absent	258 (71.5)	146 (72.6)	112 (70.0)	
Present	103 (28.5)	55 (27.4)	48 (30.0)	

Notes: Values are presented as *n* (%) or median (range), as appropriate. Percentages for variables with missing data use available-case denominators. Missing data: ECOG *n* = 25, biopsy histology *n* = 7, biopsy grade *n* = 12, and biopsy Ki-67 *n* = 8. Continuous variables were compared using the Mann–Whitney U test; categorical variables were compared using Pearson’s chi-square or exact tests, as appropriate. TC, docetaxel plus cyclophosphamide; ECOG, Eastern Cooperative Oncology Group; ER, estrogen receptor; PR, progesterone receptor; IDC, invasive ductal carcinoma; ILC, invasive lobular carcinoma; NST, no special type; LVI, lymphovascular invasion; PNI, perineural invasion.

**Table 2:** Treatment characteristics and chemotherapy delivery.

Characteristic	Overall (*n* = 361)	TC (*n* = 201)	Anthracycline–Taxane (*n* = 160)	*p* Value
**Surgical treatment**				
Breast surgery, *n* (%)				<0.001
BCS	266 (73.7)	163 (81.1)	103 (64.4)	
Mastectomy	95 (26.3)	38 (18.9)	57 (35.6)	
Axillary surgery, *n* (%)				<0.001
None	1 (0.3)	0 (0.0)	1 (0.6)	
SLNB	257 (71.2)	177 (88.1)	80 (50.0)	
ALND	103 (28.5)	24 (11.9)	79 (49.4)	
**Adjuvant treatment**				
Adjuvant radiotherapy, *n* (%)				0.389
No	38 (10.5)	24 (11.9)	14 (8.8)	
Yes	323 (89.5)	177 (88.1)	146 (91.3)	
Adjuvant endocrine therapy, *n* (%)				1.000
No	2 (0.6)	1 (0.5)	1 (0.6)	
Yes	359 (99.4)	200 (99.5)	159 (99.4)	
Endocrine treatment strategy among treated patients, *n* (%)				0.085
Aromatase inhibitor	223 (62.1)	131 (65.5)	92 (57.9)	
Tamoxifen	55 (15.3)	33 (16.5)	22 (13.8)	
OFS/LHRH + endocrine therapy	76 (21.2)	35 (17.5)	41 (25.8)	
Sequential tamoxifen-AI therapy	5 (1.4)	1 (0.5)	4 (2.5)	
**Chemotherapy delivery**				
Dose reduction, *n* (%)				0.501
No	321 (88.9)	181 (90.0)	140 (87.5)	
Yes	40 (11.1)	20 (10.0)	20 (12.5)	
Early treatment discontinuation, *n* (%)				0.441
No	345 (95.6)	194 (96.5)	151 (94.4)	
Yes	16 (4.4)	7 (3.5)	9 (5.6)	
Chemotherapy completed as planned, *n* (%)				0.467
No	17 (4.7)	8 (4.0)	9 (5.6)	
Yes	344 (95.3)	193 (96.0)	151 (94.4)	

Notes: The anthracycline–taxane group comprised ddAC+T (*n* = 45) and AC+T (*n* = 115). The anthracycline–taxane schedule consisted of four cycles of AC followed by weekly paclitaxel, typically 12 weekly doses; weekly paclitaxel administrations were not counted as conventional chemotherapy cycles. Endocrine-treatment strategy percentages were calculated among patients who received endocrine therapy (overall *n* = 359; TC *n* = 200; anthracycline–taxane *n* = 159), and the overall distribution was compared using the Fisher–Freeman–Halton exact test. Binary treatment-delivery comparisons used Fisher’s exact test. TC, docetaxel plus cyclophosphamide; ddAC+T, dose-dense doxorubicin/cyclophosphamide followed by a taxane; AC+T, doxorubicin/cyclophosphamide followed by a taxane; BCS, breast-conserving surgery; SLNB, sentinel lymph node biopsy; ALND, axillary lymph node dissection; AI, aromatase inhibitor; OFS, ovarian function suppression; LHRH, luteinizing hormone-releasing hormone.

**Table 3:** Unadjusted and overlap-weighted survival outcomes.

Outcome	Overall	TC	Anthracycline–Taxane	*p* Value
**Unadjusted survival outcomes**				
Median follow-up, months	39.0	35.0	44.8	
DFS events, *n*/*N*	12/361	3/201	9/160	
3-year DFS, %		99.0	96.6	
5-year DFS, %		97.6	90.7	
Unadjusted DFS HR (A+T vs. TC), 95% CI		Reference	2.90 (0.78–10.77)	0.096
Deaths, *n*/*N*	5/361	2/201	3/160	
3-year OS, %		99.4	99.1	
5-year OS, %		98.0	96.6	
Unadjusted OS HR (A+T vs. TC), 95% CI		Reference	1.38 (0.23–8.34)	0.725
**Primary overlap-weighted analysis (complete-case *n* = 346)**				
3-year weighted DFS, %		99.2	96.5	
Absolute 3-year DFS difference, percentage points (TC − A+T)		+2.68 (95% CI, −1.81 to +10.03)		
**Sensitivity analysis using surgical pathology (*n* = 360)**				
3-year weighted DFS, %		99.3	96.6	
Absolute 3-year DFS difference, percentage points (TC − A+T)		+2.70 (95% CI, −1.76 to +9.87)		

Notes: Median follow-up was estimated using the reverse Kaplan–Meier method. Five-year unadjusted DFS estimates should be interpreted cautiously because relatively few patients remained at risk at 60 months. HRs are presented for anthracycline–taxane chemotherapy relative to TC. Unadjusted *p* values are from log-rank tests. The primary overlap-weighted analysis included 346 patients with complete data for the biopsy-based propensity-score covariates. Absolute DFS differences are expressed as TC minus anthracycline–taxane chemotherapy. Confidence intervals for weighted absolute differences were obtained from 5000 treatment-stratified nonparametric bootstrap resamples with re-estimation of the propensity score and overlap weights in each replicate. DFS, disease-free survival; OS, overall survival; HR, hazard ratio; CI, confidence interval.

## Data Availability

The datasets generated and/or analyzed during the current study are not publicly available because they contain patient-level clinical data and are subject to institutional and ethical restrictions. Requests for access to de-identified data may be considered by the corresponding author, subject to applicable institutional and ethical approvals.

## Supplementary Materials

The following supporting information can be downloaded at https://www.mdpi.com/article/10.3390/jcm15218568/s1: Table S1: Covariate balance before and after propensity-score overlap weighting; Table S2: Sensitivity analysis using surgical-specimen tumor characteristics; Table S3: Exploratory parsimonious multivariable Cox regression analysis for disease-free survival; Table S4: Exploratory sensitivity analyses incorporating ECOG performance status and a 90-day postoperative landmark; Figure S1: Propensity-score distribution before overlap weighting in the primary biopsy-based propensity-score cohort; Figure S2: Propensity-score distribution after overlap weighting in the primary biopsy-based propensity-score cohort; Figure S3: Unadjusted overall survival in the full study cohort.
