# Supplementary material for: Adjuvant Docetaxel–Cyclophosphamide Versus Anthracycline–Taxane Chemotherapy in Stage I–III HR-Positive/HER2-Negative Breast Cancer: A Real-World Propensity Score Overlap-Weighted Cohort Study
Source: J Clin Med. 2026 Nov 3;15(21):8568. doi: 10.3390/jcm15218568 (PMC13763730; doi:10.3390/jcm15218568)
Supplement: Supplementary file 1 [file jcm-15-08568-s001.zip › jcm-4651327-supplementary.pdf]

Supplementary Materials

Supplementary Table S1. Covariate balance before and after propensity-score overlap weighting

Covariate	Absolute SMD before weighting	Absolute SMD after overlap weighting
Age at diagnosis	0.208	<0.001
Year of diagnosis	0.363	<0.001
Post/perimenopausal status	0.168	<0.001
Tumor size	0.501	<0.001
Nodal burden: N1/N1mi	0.734	<0.001
Nodal burden: N2–N3	0.758	<0.001
Biopsy grade 2	0.134	<0.001
Biopsy grade 3	0.146	<0.001
Biopsy Ki-67	0.209	<0.001
Biopsy histology: ILC	0.096	<0.001
Biopsy histology: NOS/NST	0.051	<0.001
Biopsy histology: mixed	0.018	<0.001
Biopsy histology: special type	0.040	<0.001
Lymphovascular invasion	0.242	<0.001

Note: Absolute standardized mean differences (SMDs) are shown. An absolute SMD <0.10 was considered indicative of adequate balance. The primary propensity-score cohort included 346 patients (TC, n=195; anthracycline–taxane, n=151). After overlap weighting, all modeled covariates showed excellent balance (maximum absolute SMD <0.001). Values below 0.001 reflect the exact mean-balance property of logistic-regression overlap weights for modeled covariates, with displayed values rounded to three decimals. Effective sample size (ESS), calculated as $(\Sigma w)^2/\Sigma(w^2)$, was 81.4 for the TC group and 80.2 for the anthracycline–taxane group (overall ESS, approximately 161.6).

Supplementary Table S2. Sensitivity analysis using surgical-specimen tumor characteristics

Outcome	TC	Anthracycline–taxane	Effect estimate
Analysis cohort, n	200	160	
3-year weighted DFS, %	99.3	96.6	+2.70 pp (95% CI -1.76 to +9.87)

Note: The sensitivity propensity-score model replaced biopsy grade, Ki-67, and histology with the corresponding surgical-specimen variables while retaining the remaining propensity-score covariates. The analysis included 360 patients (TC, n=200; anthracycline–taxane, n=160). The effect estimate is expressed as TC minus anthracycline–taxane chemotherapy. DFS, disease-free survival; CI, confidence interval; pp, percentage points.

Supplementary Table S3. Exploratory parsimonious multivariable Cox regression analysis for disease-free survival

Variable	Adjusted HR	95% CI	p value
Anthracycline–taxane vs TC	1.32	0.25–7.06	0.745
Maximum tumor size, per 10-mm increase	1.06	0.80–1.40	0.704
Nodal burden: N1/N1mi vs N0	2.58	0.39–17.03	0.325
Nodal burden: N2–N3 vs N0	4.58	0.53–39.33	0.166

Note: Because only 12 DFS events occurred, the multivariable model was intentionally restricted to treatment group, maximum tumor size, and pathologic nodal burden to reduce the risk of overfitting. The analysis was exploratory and complementary to the primary propensity-score overlap-weighted analysis. HR, hazard ratio; CI, confidence interval; DFS, disease-free survival; TC, docetaxel–cyclophosphamide.

Supplementary Table S4. Exploratory sensitivity analyses incorporating ECOG performance status and a 90-day postoperative landmark

Analysis	Cohort n (TC / A+T)	3-year DFS TC, %	3-year DFS A+T, %	Difference, pp (95% CI)
ECOG-adjusted overlap weighting	323 (195 / 128)	99.7	95.5	+4.14 (−0.99 to +13.44)
90-day landmark overlap weighting	337 (193 / 144)	99.1	94.0	+5.14 (−1.06 to +14.34)

Note: Both analyses used the biopsy-based propensity-score covariates from the primary analysis and 5,000 treatment-stratified nonparametric bootstrap resamples with re-estimation of the propensity score and overlap weights in each resample. The ECOG analysis additionally included ECOG status (0, 1, or 2) and was restricted to complete ECOG cases (11 DFS events); ECOG was missing only in the anthracycline–taxane group. The 90-day landmark analysis included patients with complete biopsy-based covariates who had initiated adjuvant chemotherapy by postoperative day 90 and remained event-free at that landmark (12 DFS events); survival time was measured from postoperative day 90 rather than from surgery. Nine patients from the primary complete-case cohort started chemotherapy after day 90 and were excluded from the landmark analysis. Both analyses were exploratory and their results are not directly comparable because of differences in inclusion criteria and time origins. Difference = TC minus anthracycline–taxane; DFS, disease-free survival; CI, confidence interval; pp, percentage points; ECOG, Eastern Cooperative Oncology Group.

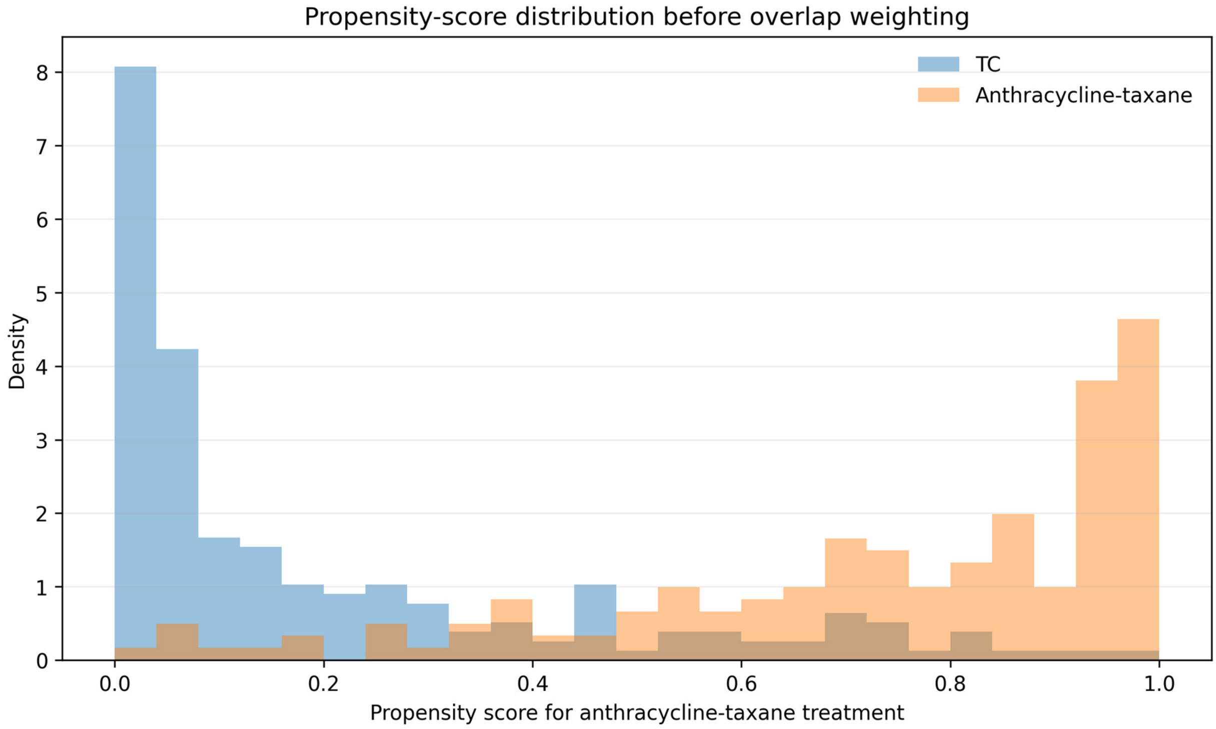

Supplementary Figure S1. Propensity-score distribution before overlap weighting in the primary biopsy-based propensity-score cohort (n=346). The marked separation between treatment groups illustrates substantial differences in the probability of receiving anthracycline–taxane chemotherapy before weighting.

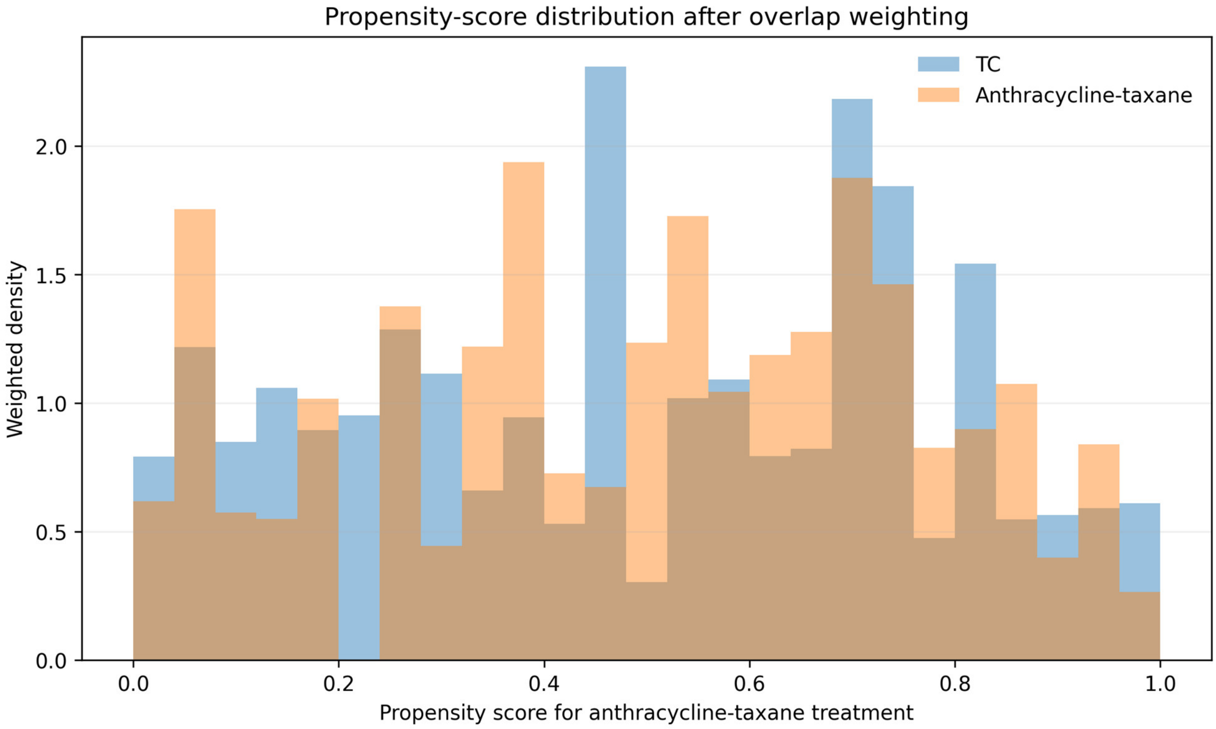

Supplementary Figure S2. Propensity-score distribution after overlap weighting in the primary biopsy-based propensity-score cohort. The weighted distributions are substantially aligned, consistent with the excellent covariate balance demonstrated by the standardized mean differences.

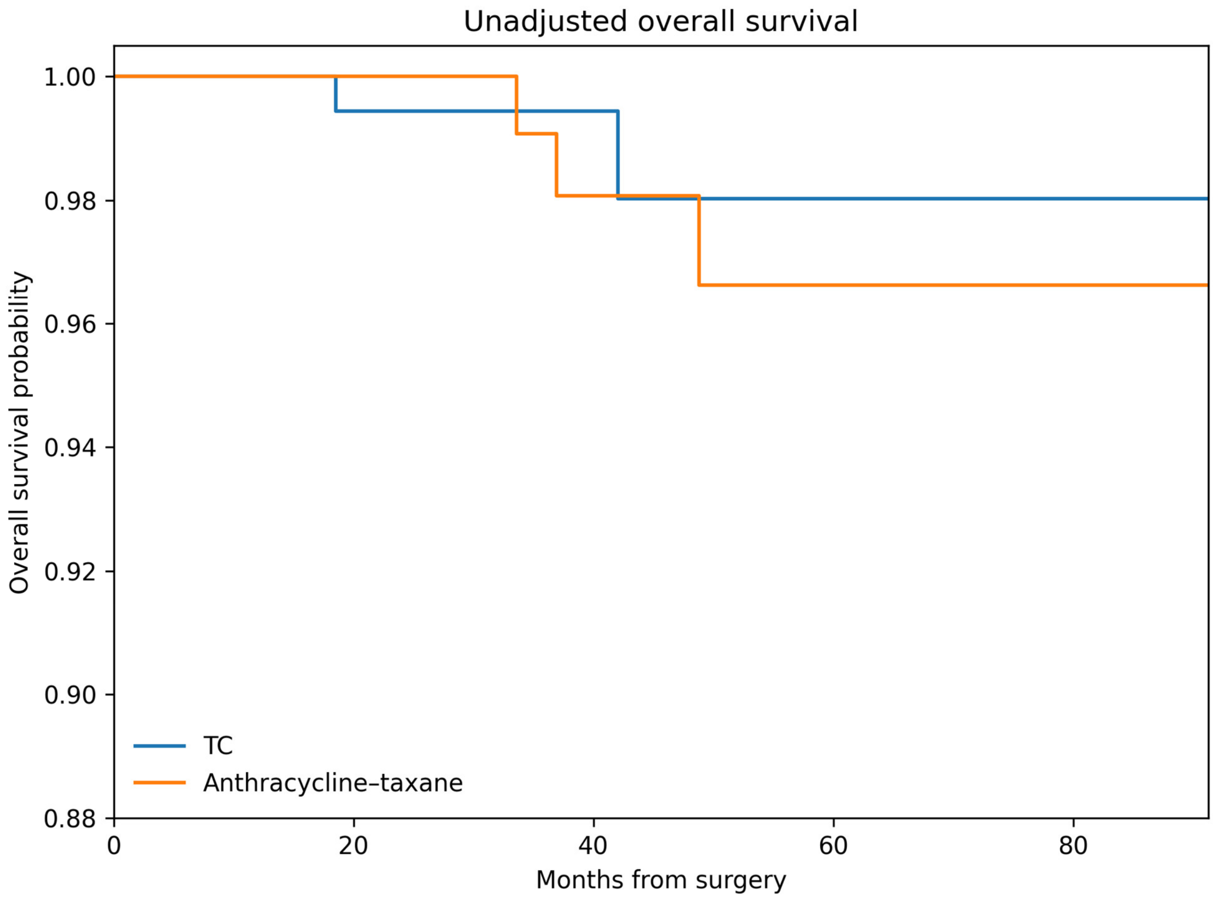

Supplementary Figure S3. Unadjusted overall survival in the full study cohort (TC, n=201; anthracycline–taxane, n=160). Five deaths occurred during follow-up. The unadjusted hazard ratio for anthracycline–taxane chemotherapy versus TC was 1.38 (95% CI, 0.23–8.34; log-rank p=0.725).
